# Design of Phosphonium-Type Zwitterion as an Additive to Improve Saturated Water Content of Phase-Separated Ionic Liquid from Aqueous Phase toward Reversible Extraction of Proteins

**DOI:** 10.3390/ijms140918350

**Published:** 2013-09-05

**Authors:** Yoritsugu Ito, Yuki Kohno, Nobuhumi Nakamura, Hiroyuki Ohno

**Affiliations:** 1 Department of Biotechnology, Tokyo University of Agriculture and Technology, 2-24-16, Naka-cho, Koganei, Tokyo 184-8588, Japan; E-Mails: yito4@mmm.com (Y.I.); Yuki.Kono@colorado.edu (Y.K.); nobu1@cc.tuat.ac.jp (N.N.); 2 Functional Ionic Liquid Laboratories, Graduate School of Engineering, Tokyo University of Agriculture and Technology, 2-24-16, Naka-cho, Koganei, Tokyo 184-8588, Japan; 3 Japan Science and technology Agency (JST), Core Research for Evolutional Science and Technology (CREST), Chiyoda, Tokyo 102-0076, Japan

**Keywords:** ionic liquid/water biphasic system, protein extraction, zwitterion, hydrophobicity, recovering

## Abstract

We designed phosphonium-type zwitterion (ZI) to control the saturated water content of separated ionic liquid (IL) phase in the hydrophobic IL/water biphasic systems. The saturated water content of separated IL phase, 1-butyl-3-methyimidazolium bis(trifluoromethanesulfonyl)imide, was considerably improved from 0.4 wt% to 62.8 wt% by adding *N*,*N*,*N*-tripentyl-4-sulfonyl-1-butanephosphonium-type ZI (P_555_C4S). In addition, the maximum water content decreased from 62.8 wt% to 34.1 wt% by increasing KH_2_PO_4_/K_2_HPO_4_ salt content in upper aqueous phosphate buffer phase. *Horse heart cytochrome c* (cyt.*c*) was dissolved selectively in IL phase by improving the water content of IL phase, and spectroscopic analysis revealed that the dissolved cyt.*c* retained its higher ordered structure. Furthermore, cyt. *c* dissolved in IL phase was re-extracted again from IL phase to aqueous phase by increasing the concentration of inorganic salts of the buffer solution.

## 1. Introduction

Ionic liquids (ILs) are organic molten salts with very low melting temperature [1–3]. It is not so difficult to tune a wide variety of liquid properties including polarity [4], viscosity [5], and hydrophobicity [6] by appropriate combination of cation and anion species. There are a growing number of articles on aqueous biphasic systems (ABSs) based on ILs [7]. ABS offers an attractive alternative to conventional extraction methods for the separation of numerous kinds of materials such as biomolecules. Previous ABSs were mainly composed of two different polymers or a polymer and aqueous salt solution. These ABSs have been used for product recovery and downstream processing in biotechnology. It was shown that not only polymers like poly(ethylene glycol) and dextran but also ILs in combination with a kosmotropic inorganic salt are able to form ABS [8]. Some ILs having hydrophobic ions easily form liquid/liquid biphases after mixing with water. These hydrophobic IL/water biphasic systems have been broadly studied and applied as novel energy-saving processes for extraction, separation, and condensation of biopolymers. More specifically, solubilization and stabilization of proteins in separated hydrophobic IL phase rather than aqueous phase would lead to the interesting application of ILs as continuous enzymatic reaction and/or product separation systems. It would be very convenient to separate enzymes from reaction products dissolved in an aqueous phase after the enzymatic reaction. However, most enzymes are not soluble in hydrophobic ILs. Some methods exist to dissolve proteins in hydrophobic ILs, such as modification of proteins with amphiphilic polymers [9,10], addition of crown ethers [11], and formation of aqueous microemulsion droplets in a hydrophobic IL [12].

According to our previous results on hydrated ILs [13–15], it would be essential to improve the hydrated state of these hydrophobic ILs for stable dissolution of proteins. However, there was no report on the successive control of water content in hydrophobic ILs until today. To control the water content of the hydrophobic ILs, one can design component ions of ILs with suitable hydrophilicity [16,17]. By this method, it is not easy to control the desirable amount of saturated water in the ILs. Other methods include simple mixing of some hydrated ILs. Mixing two or more ILs is much easier for the fine-tuning of the hydrophilicity of ILs than structural design of the ILs. However, there is always a fear of the ion exchange reaction among the mixed ions that would lead to unexpected change in the physico-chemical properties of pristine ILs. Recently, we proposed zwitterions (ZIs) as additives suitable for controlling the water content of hydrophobic ILs [18]. ZIs should prevent ion exchange in the IL mixtures, because their component cations and anions are both covalently tethered. In our previous study, saturated water content of [C_4_mim][Tf_2_N] was improved from 0.4 wt% to 17.8 wt% by adding hydrated 3-(1-butyl-3-imidazolio)propanesulfonate-type ZI (C_4_Im3S) in appropriate amounts [18]. However the saturated water content of IL phase showed little increase after adding excess amount of water to obtain biphasic systems. This was because C_4_Im3S was partitioned to aqueous phase due to high hydrophilicity of C_4_Im3S. When suitably hydrated ZIs were partitioned selectively in hydrophobic IL phase, the resulting ZI would be an effective additive to improve the water content in the separated IL phase. In the present study, we have designed ZIs with suitable hydrophilicity to accomplish both improving hydrated state and partitioning in the separated IL phase. The distribution of cyt.*c* in the hydrophobic IL/water mixture and re-extraction of cyt.*c* from IL phase to aqueous phase have been also analyzed.

## 2. Results and Discussion

### 2.1. Control of Saturated Water Content of Separated IL Phase by Mixing Phosphonium-Type ZI

**Figure 1:**
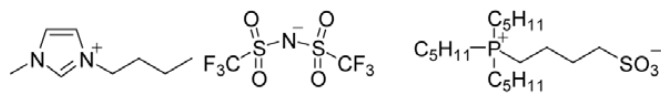
Structure of ionic liquid (IL) and zwitterion (ZI) in this study; **left**: [C_4_mim][Tf_2_N]; **right**: P_555_C4S.

**Figure 2:**
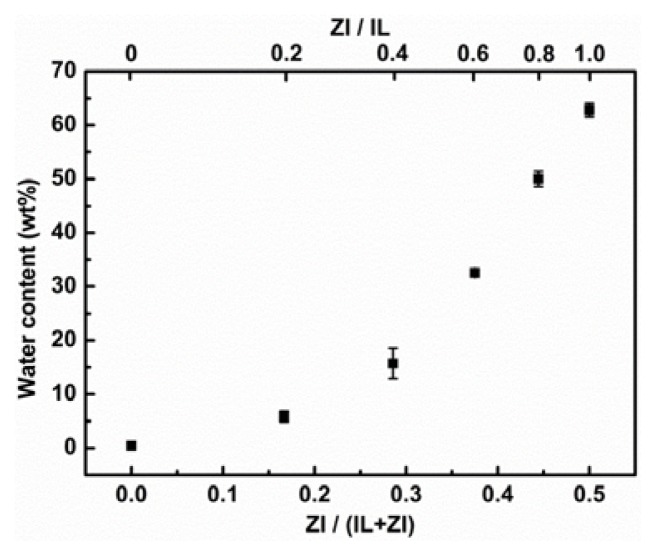
Relation between water content of IL phase and fraction of ZI in IL/ZI mixtures.

Figure 1 shows both ZI and IL prepared and used in this study. *N*,*N*,*N*-Tripentyl-4-sulfonyl-1-butanephosphonium (P_555_C4S) was mixed with equimolar [C_4_mim][Tf_2_N], then pure water was added to [C_4_mim][Tf_2_N]/P_555_C4S mixture as 1:10 by volume. The resulting mixture was not phase-separated but homogeneously mixed. Instead, when phosphate buffer (100 mM, pH 7.0) was added to [C_4_mim][Tf_2_N]/P_555_C4S mixture as 1:10 by volume, the resulting mixture was found to form liquid/liquid phase separation. Then different amount of P_555_C4S was mixed with [C_4_mim][Tf_2_N] and phosphate buffer (100 mM, pH 7.0). The resulting solutions were stirred vigorously and stored. The saturated water content of phase separated IL phase was then determined by Karl-Fischer titration method. The saturated water content of the [C_4_mim][Tf_2_N] phase increased with the amount of the P_555_C4S as shown in Figure 2. This result suggests that the saturated water content of hydrophobic ILs can be controlled by the added amount of P_555_C4S. As seen in Figure 2, addition of P_555_C4S improves the saturated water content of [C_4_mim][Tf_2_N] from 0.4 wt% to 62.8 wt%. The water content of separated [C_4_mim][Tf_2_N] phase without P_555_C4S was comparable to the previously reported value [19]. It follows that suitably hydrophobic P_555_C4S comprising phosphonium cation with long alkyl chains and sulfonate anions to increase saturated water content of hydrophobic IL phase.

### 2.2. Effect of K_2_HPO_4_/KH_2_PO_4_ Concentration in Aqueous Phase on the Water Content of IL Phase

**Figure 3:**
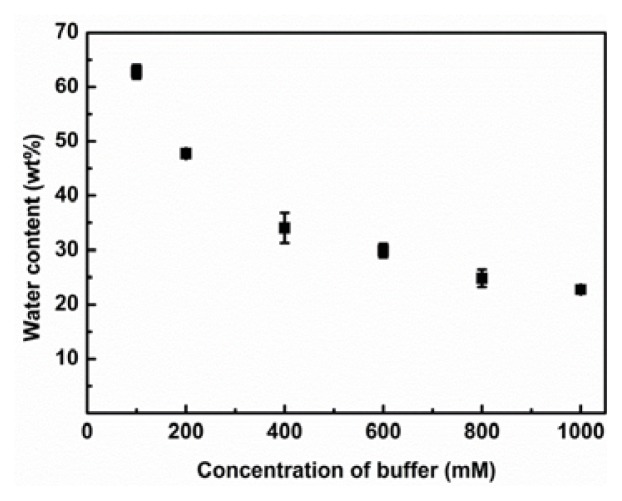
Relationship between water content of IL phase and concentration of K_2_HPO_4_/KH_2_PO_4_ salts in buffer solution.

We next studied the effect of concentration of K_2_HPO_4_/KH_2_PO_4_ salts in buffer solution added to mixture on the saturated water content of hydrophobic IL phase. An equimolar P_555_C4S was mixed with [C_4_mim][Tf_2_N], and phosphate buffers with different concentration of inorganic salts (from 100 to 1000 mM, pH 7.0) were added to [C_4_mim][Tf_2_N]/P_555_C4S mixture as 1:10 by volume. The resulting solutions were stirred vigorously. The saturated water content of phase separated IL phase was also determined using the same procedure as describe above. The saturated water content of the [C_4_mim][Tf_2_N] phase decreased with increasing the amount of inorganic salts (Figure 3). The decrease of saturated water content of IL phase attributed to concentrated inorganic salts strongly interacting water molecules. Rogers *et al.* reported the pioneering research on formation of ABS by the addition of inorganic salts to aqueous solution of hydrophilic ionic liquids [8]. They firstly demonstrated the formation of ABS between hydrophilic [C_4_mim]Cl-rich upper phase and inorganic-salt-rich bottom phase based on the salting-out phenomena of the inorganic salts [8]. Coutinho *et al.* reported effects of both IL structure and salting-out species (inorganic salts, organic salts, and so on) on the phase equilibria of ABSs [7,20–22]. They concluded that the necessary factor for formation of ABS comprising ionic liquids and conventional salts was the salting-out effect resulting in the creation of water-ion complexes that cause the dehydration of the solute and the increase in the surface tension of the cavity in the aqueous media. It is plausible that the decrease of the saturated water content of [C_4_mim][Tf_2_N] by adding phosphate buffer was derived from the salting-out effect of K_2_HPO_4_/KH_2_PO_4_ salts.

### 2.3. Extraction of Cytochrome *c* from Aqueous Phase to IL Phase by Mixing Phosphonium-Type ZI

**Figure 4:**
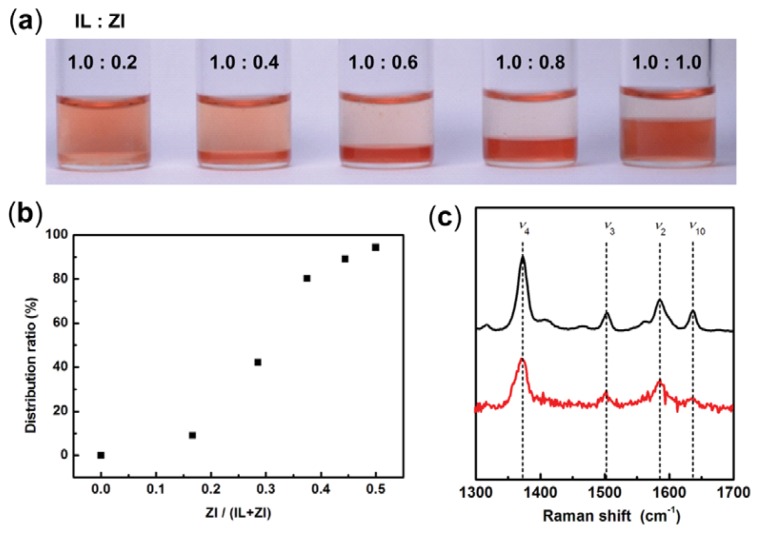
Extraction of cyt.*c* from aqueous to IL phase by mixing P_555_C4S-type ZI. (**a**) Photograph of [C_4_mim][Tf_2_N]/P_555_C4S/buffer mixture that different amount of P_555_C4S added; (**b**) Relation between distribution ratio of cyt.*c* and mole fraction of ZI in IL/ZI mixtures; (**c**) Resonance Raman spectra of cyt.*c* in phosphate buffer (black line), and cyt.*c* in IL phase of [C_4_mim][Tf_2_N]/P_555_C4S/buffer mixture (red line).

**Figure 5:**
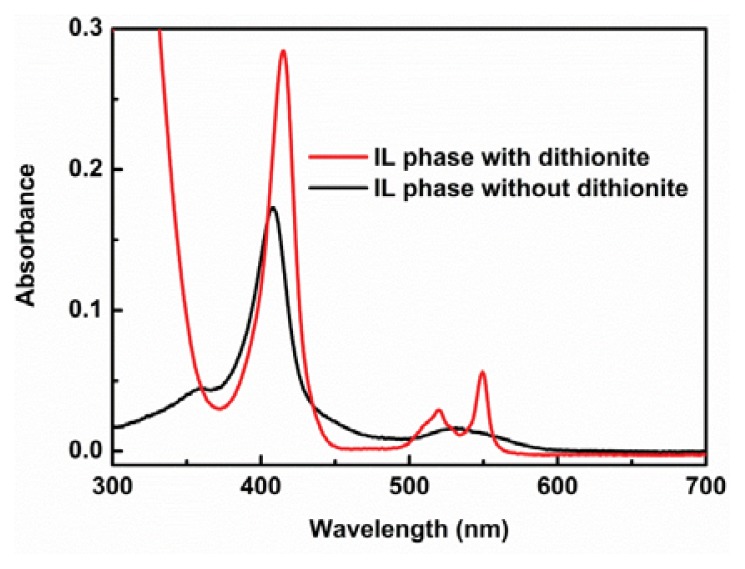
UV spectra of cyt.*c* extracted to IL phase before (black line) and after (red line) adding excess amount of dithionite. The [C_4_mim][Tf_2_N]/P_555_C4S ratio was 1:1.

Solubility of *horse heart cytochrome c* (cyt.*c*) in our [C_4_mim][Tf_2_N]/P_555_C4S/buffer mixture was analyzed to clarify the effect of ZI concentration. Both [C_4_mim][Tf_2_N] and P_555_C4S were mixed as the mixing ratio of [C_4_mim][Tf_2_N]/P_555_C4S from 1.0:0.2 to 1.0:1.0. Cyt.*c* was dissolved into phosphate buffer (100 mM, pH 7.0) and added to [C_4_mim][Tf_2_N]/P_555_C4S mixture. The resulting solutions were stirred vigorously. Figure 4a shows a photograph of cyt.*c* in the mixtures. The distribution ratio (*D*) was calculated from the maximum absorbance of the Soret band around 408.0 nm of cyt.*c* in each phase of [C_4_mim][Tf_2_N]/P_555_C4S/buffer mixture. As seen in Figure 4b, the *D* value was found to increase with increasing the saturated water content of IL phase by adding P_555_C4S. When an equimolar amount of P_555_C4S was mixed with [C_4_mim][Tf_2_N], the *D* value reached up to 94%. Since cyt.*c* was not soluble in [C_4_mim][Tf_2_N] neither pure nor buffer-saturated, it is confirmed that the P_555_C4S facilitated the dissolution of cyt.*c* in the [C_4_mim][Tf_2_N]. Resonance Raman (RR) spectroscopy of cyt.*c* in the mixture was then undertaken in order to determine the environment of the heme vicinity. Several relevant modes observed in the RR spectra are sensitive both to axial coordination and the spin state of the iron ion of heme [23]. In the RR spectrum of cyt.*c* in buffer solution at pH 7.0, the ν_4_ band around 1372 cm^−1^ represents the valency of the heme iron. The ν_2_ (1586 cm^−1^) and ν_3_ (1502 cm ^−1^) bands are both helpful in determining both the coordination state and the spin state, and these bands indicated that the heme iron ion was in a six-coordinated low spin state. Figure 4c shows the RR spectra of cyt.*c* in phosphate buffer (black line), and cyt.*c* in IL phase of [C_4_mim][Tf_2_N]/P_555_C4S/buffer mixture (ratio of IL:ZI was 1:1) (red line). The RR spectrum of cyt.*c* in the [C_4_mim][Tf_2_N]/P_555_C4S/buffer mixture included intense bands at 1372 cm^−1^ (ν_4_), 1502 cm^−1^ (ν_3_), 1586 cm^−1^ (ν_2_), and 1634 cm^−1^ (ν_10_), which indicate that the central iron ion of the heme complex is in a oxidized and six-coordinated low spin state. These characteristic bands are similar to these for cyt.*c* in buffer solution, suggesting that the dissolution of cyt.*c* in the [C_4_mim][Tf_2_N]/P_555_C4S/buffer mixture did not give rise to a drastic change in the heme vicinity. Furthermore, spectroscopic analysis has been undertaken to determine the dissolved state and stability of cyt.*c* in the [C_4_mim][Tf_2_N]/P_555_C4S/buffer mixture. Since cyt.*c* is known as a typical redox-active protein, the activity of the cyt.*c* in the IL phase is easily detectable by analyzing the redox response. The IL phase containing cyt.*c* was pipetted out and UV-vis spectra of the IL phase was measured. At the initial stage, both strong Soret band and broad Q-band were observed at 408 and 530 nm, respectively (Figure 5, black line). These bands indicate that cyt.*c* exists as an oxidized state in the IL phase similar to that in an aqueous buffer solution. When a small excess amount of sodium dithionite was added as a reducing agent to the IL phase, small amount of water was drained from the IL phase but cyt.*c* was still dissolved in the IL phase. Then UV-vis spectrum of the IL phase was also measured to find the reduced cyt.*c* (Figure 5, red line). The spectrum showed a sharp α- and β-band at 550 nm and 521 nm, respectively in a Q-band region and a Soret band at 415 nm, typically seen in reduced cyt.*c* in an aqueous buffer solution. Relatively large absorbance of the reduced cyt.*c* as shown in Figure 5 was due to the increased concentration of cyt.*c* by losing small amount of water as described above. This means that cyt.*c* retains the redox activity even after treated with the ILs. Both solubilization and stabilization of cyt.*c* in [C_4_mim][Tf_2_N] phase are accomplished by adding P_555_C4S without damaging its functionality.

### 2.4. Effect of K_2_HPO_4_/KH_2_PO_4_ Concentration in Aqueous Phase on the Distribution Ratio of cyt.*c*

**Figure 6:**
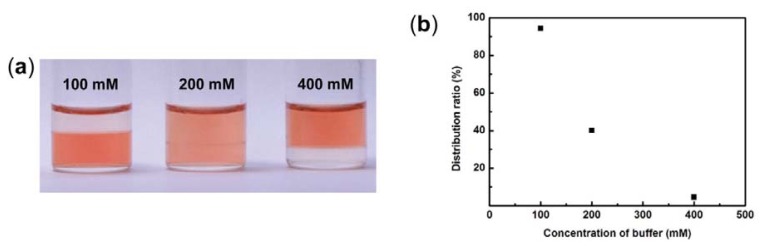
Control of *D* value by mixing different amounts of K_2_HPO_4_/KH_2_PO_4_ salts. (**a**) Photograph of [C_4_mim][Tf_2_N]/P_555_C4S/buffer mixture that different concentration of phosphate buffer used. Upper phase: aqueous buffer solution, lower phase: IL rich phase; (**b**) Relationship between distribution ratio of cyt.*c* in IL phase and concentration of buffer solution.

As mentioned above, the saturated water content of IL phase decreased upon increasing the amount of K_2_HPO_4_/KH_2_PO_4_ salts in an aqueous buffer solution phase (Figure 3). This result suggested that the *D* value of cyt.*c* was changed by the amount of the added inorganic salts. We then studied the effect of concentration of K_2_HPO_4_/KH_2_PO_4_ salts in buffer solution on the *D* value of cyt.*c* in the [C_4_mim][Tf_2_N]/P_555_C4S/buffer mixture. Both [C_4_mim][Tf_2_N] and P_555_C4S were mixed with the mixing ratio of [C_4_mim][Tf_2_N]/P_555_C4S of 1:1. Cyt.*c* was then dissolved in phosphate buffer with different concentration of inorganic salts (100, 200, and 400 mM, pH 7.0). These aqueous solutions (1 mg mL^−1^) of cyt.*c* were added to [C_4_mim][Tf_2_N]/P_555_C4S mixture as 1:10 by volume of IL:buffer solution. Figure 6a shows a photograph of cyt.*c* in the mixtures with different concentration of K_2_HPO_4_/KH_2_PO_4_ salts. The *D* value of cyt.*c* in the [C_4_mim][Tf_2_N]/P_555_C4S/buffer mixture was determined by the same procedure as described above. The *D* value of cyt.*c* in the mixture decreased with the amount of K_2_HPO_4_/KH_2_PO_4_ salts of buffer solution (Figure 6b). In the case of 400 mM phosphate buffer solution, almost all cyt.*c* remained in the aqueous buffer solution phase rather than IL phase. This result suggested that the saturated water content of IL phase strongly affected to dissolution of cyt.*c* to IL phase.

### 2.5. Construction of Reversible Extraction Process for cyt.*c* by [C_4_mim][Tf_2_N]/P_555_C4S/Buffer Mixture

**Figure 7:**
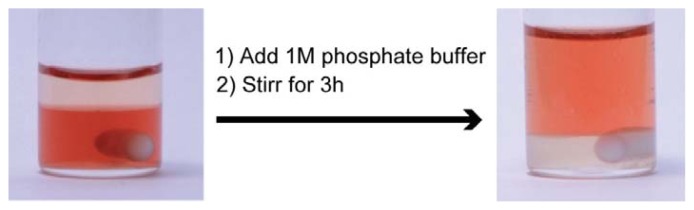
Reverse extraction process of cyt.*c* from IL phase to aqueous phase by controlling water content of IL phase.

In previous sections, it was confirmed that the saturated water content of IL phase increased by adding P_555_C4S and decreased by using high concentration buffer. Furthermore, the saturated water content of IL phase strongly affected to the *D* value of cyt.*c* in the [C_4_mim][Tf_2_N]/P_555_C4S/buffer mixture. From the obtained results, we speculated that re-extraction of Cyt.*c* from IL phase to aqueous phase should be facilitated by adding concentrated inorganic salt solution. Then we investigated the partition behavior of cyt.*c* between IL phase and aqueous buffer phase after mixing different concentration of inorganic salt solutions. [C_4_mim][Tf_2_N] and P_555_C4S were mixed as the mixing ratio of [C_4_mim][Tf_2_N]:P_555_C4S were 1:1. Cyt.*c* was then dissolved into phosphate buffer (100 mM, pH 7.0). The aqueous solution of cyt.*c* (2 mg mL^−1^) were added [C_4_mim][Tf_2_N]/P_555_C4S mixture as 1:10 by volume of IL:buffer solution. After stirring the solution and being left until the phases became clear (Figure 7 left), 1.0 M buffer solution was then added to the solution to reach the concentration of upper inorganic salt solution to 400 mM. It is easily confirmed by naked eyes that cyt.*c* has transferred from IL phase to buffer phase by controlling the concentration of inorganic salt of buffer solution (Figure 7 right). The environment of heme vicinity and redox activity of cyt.*c* extracted from IL phase to buffer phase in the mixture was also determined by the same method described above, and confirmed to retain their initial states in an aqueous buffer solution. Cyt.*c* was accordingly re-extracted from IL phase to buffer phase by controlling the saturated water content of IL phase with concentration of inorganic salt of buffer solution, without significant change of higher ordered structure.

In 2003, Rogers *et al.* reported the IL-based ABS by adding inorganic salts to aqueous solution of hydrophilic ILs [8]. Since then, considerable efforts have been made to construct various ABSs with hydrophilic ILs and inorganic salts as a salting-out agent [7]. However, very few reports can be found in the literature on the extraction of proteins and enzymes using hydrophobic ILs that easily undergo phase separation by adding water. This is probably due to the limited solubility of proteins in most hydrophobic ILs. In our proposed ABS systems, the water content of separated hydrophobic IL phase can be finely controlled by adding adequate amounts of ZI with suitable hydrophobicity. In addition, reversible partition of proteins was facilitated depending on the concentration of inorganic salts. This result strongly suggests that adding ZIs would provide novel bio-related engineering process including continuous enzymatic reaction systems and downstream processes by exploiting hydrophobic IL/water biphasic systems.

## 3. Experimental Section

### 3.1. Materials

1-Methylimidazole, 1-bromobutane and 1,4-butanesultone were purchased from Tokyo Chemical Industry Co. Ltd. (Tokyo, Japan). Dipotassium hydrogen phosphate, acetonitrile, diethylether, dichloromethane, toluene, ethyl acetate, methanol were purchased from Kanto Chemical Co. Inc. (Tokyo, Japan). Potassium dihydrogen phosphate was purchased from Wako Pure Chemical Co. Ltd. (Osaka, Japan). Lithium bis(trifluoromethanesulfonyl)imide was the gift from Sumitomo 3M Ltd (Tokyo, Japan). Cytochrome *c* from *horse heart* and aluminum oxide were purchased from Sigma-Aldrich Japan Co. (Tokyo, Japan). Tripentylphosphine was donated from Hokko Chemical Industry Co. Ltd. (Tokyo, Japan). Both 1-methylimidazole and 1-bromobutane were distilled before use.

### 3.2. Preparation of ILs and ZI

#### 3.2.1. 1-Butyl-3-Methylimidazolium Bromide ([C_4_mim]Br)

A zwitterion (ZI) and an ionic liquid (IL) used in this study were synthesized as reported previously [24]. We prepared 1-butyl-3-methylimidazolium bromide ([C_4_mim]Br) as follows. 1-Methylimidazole was dissolved into acetonitrile, and mixed with 1-bromobutane under dry nitrogen. The resulting solution was stirred for 24 h at room temperature. After removal of acetonitrile by evaporation, the residual liquid was repeatedly washed with excess amounts of anhydrous diethyl ether. After evaporation of the lower phase, resultant liquid was dried *in vacuo* at room temperature for 24 h. A structure of [C_4_mim]Br was confirmed by ^1^H-NMR spectroscopy. These spectra were observed using a JEOL ECX-400. ^1^H-NMR (400MHz, CDCl_3_, δ/ppm relative to TMS): 0.96 (3H, t, *J* = 14.6, N-(CH_2_) _3_*CH_3_*), 1.39 (2H, m, *J* = 37.6, N-(CH_2_)_2_*CH_2_*CH_3_), 1.92 (2H, m, *J* = 30.2, N-CH_2_*CH_2_* CH_2_CH_3_), 4.14 (3H, s, N-*CH_3_*), 4.36 (2H, t, *J* = 14.7, N-*CH_2_*(CH_2_)_2_CH_3_), 7.61 (1H, s, imidazolium cation), 7.73 (1H, s, imidazolium cation), 10.25 (1H, s, imidazolium cation).

#### 3.2.2. 1-Butyl-3-Methylimidazolium Bis(trifluoromethanesulfonyl)imide ([C_4_mim][Tf_2_N])

We then prepared 1-butyl-3-methylimidazolium bis(trifluoromethanesulfonyl)imide ([C_4_mim][Tf_2_N]) as follows. First, [C_4_mim]Br and lithium bis(trifluoromethanesulfonyl)imide were individually dissolved in water, and the resulting solutions were mixed under dry nitrogen gas atmosphere. The resulting solution was further stirred for 24 h at room temperature. After dissolution to dichloromethane, the resulting solution was repeatedly washed with excess amounts of water. After evaporation of the dichloromethane phase, resultant liquid was dried *in vacuo* at room temperature for 24 h. The structure of [C_4_mim]Br was confirmed by ^1^H-NMR spectra. These spectra were observed using a JEOL ECX-400. ^1^H-NMR (400 MHz, CDCl_3_, δ/ppm relative to TMS): 0.96 (3H, t, *J* = 15.1, N-(CH_2_) _3_*CH_3_*), 1.36 (2H, m, *J* = 37.1, N-(CH_2_)_2_*CH_2_*CH_3_), 1.85 (2H, m, *J* = 30.2, N-CH_2_*CH_2_*CH_2_CH_3_), 3.93 (3H, s, N-*CH_3_*), 4.17 (2H, t, *J* = 15.1, N-*CH_2_*(CH_2_)_2_CH_3_), 7.27 (1H, s, imidazolium cation), 7.31 (1H, s, imidazolium cation), 8.73 (1H, s, imidazolium cation).

#### 3.2.3. *N*,*N*,*N*-Tripentyl-4-Sulfonyl-1-Butanephosphonium (P_555_C4S)

We next prepared *N*,*N*,*N*-tripentyl-4-sulfonyl-1-butanephosphonium (P_555_C4S) as follows. Tripentylphosphine and 1,4-butanesultone were dissolved into toluene, and the resulting solutions were mixed under dry nitrogen gas atmosphere. The resulting solution was stirred for three days at 100 °C. After removal of toluene by evaporation, the residual liquid was repeatedly washed with excess amounts of anhydrous diethylether. A resultant solid was dissolved in dichloromethane, and the solution was passed through a column filled with aluminum oxide. It was then further purified by recrystallization from ethylacetate, and resultant white powder was dried *in vacuo* at room temperature for 24 h. Structure of P_555_C4S was confirmed by ^1^H-NMR spectra. ^1^H-NMR (400 MHz, CDCl_3_, δ/ppm relative to TMS): 0.92 (9H, t, P-(CH_2_)_4_*CH_3_*), 1.37 (6H, m, P-(CH_2_)_2_*CH_2_*CH_2_CH_3_), 1.48 (12H, m, P-CH_2_*CH_2_*CH_2_*CH_2_*CH_3_), 1.76 (2H, m, P-CH_2_*CH_2_*(CH_2_)_2_SO_3_), 2.00 (2H, t, P-*CH_2_*(CH_2_)_3_SO_3_), 2.22 (6H, m, P-*CH_2_*(CH_2_)_3_CH_3_), 2.47 (2H, m, P-(CH_2_)_2_*CH_2_*CH_2_SO_3_), 2.87 (2H, t, P-(CH_2_)_3_*CH_2_*SO_3_).

### 3.3. Water Content of Hydrophobic IL Phase after Mixing with P_555_C4S

Both [C_4_mim][Tf_2_N] and P_555_C4S were mixed at the mixing ratio of [C_4_mim][Tf_2_N]/P_555_C4S from 1:0.2 to 1:1. Phosphate buffer (100 mM, pH 7.0) was added to [C_4_mim][Tf_2_N]/P_555_C4S mixture as 1:10 by volume, and the resulting solutions were stirred vigorously. The saturated water content of phase separated IL phases was determined by Karl-Fischer titration method (Kyoto Electrons; MKC-520N) after dilution of IL phase by dehydrated methanol.

### 3.4. Control of Water Content of Hydrophobic IL Phase by Adding Phosphate Buffers

P_555_C4S was mixed with equimolar amount of [C_4_mim][Tf_2_N], then phosphate buffer with different concentration of K_2_HPO_4_/KH_2_PO_4_ salts (from 0.1 to 1.0 M, pH 7.0) were added to [C_4_mim][Tf_2_N]/P_555_C4S mixture as 1:10 by volume of IL:buffer solution. The resulting solutions were stirred vigorously. The saturated water content of phase separated IL phases was determined by using the same procedure as describe above.

### 3.5. Distribution Ratio of cyt.*c* in Hydrophobic IL/Water Biphasic System

[C_4_mim][Tf_2_N] and P_555_C4S were mixed at the mixing ratio of from 1:0.2 to 1.0:1.0. Cytochrome *c* from *horse heart* was dissolved into phosphate buffer with different concentration of K_2_HPO_4_/KH_2_PO_4_ salts (100, 200, and 400 mM, pH 7.0). These aqueous solutions (1 mg mL^−1^) of cyt.*c* were added to [C_4_mim][Tf_2_N]/P_555_C4S mixtures as 1:10 by volume of IL:buffer solution. The resulting solutions were stirred vigorously. The distribution ratio (*D*) was calculated from the absorbance of cyt.*c* in each phase of [C_4_mim][Tf_2_N]/P_555_C4S/buffer mixture, with the equation; *D* = (*Abs*_IL_ × *V*_IL_)/(*Abs*_IL_ × *V*_IL_ + *Abs*_buffer_ × *V*_buffer_), where *Abs*_IL_ and *Abs*_buffer_ corresponded to the absorbance of IL phase and buffer phase, respectively, and *V*_IL_ and *V*_buffer_ corresponded to the volume of IL phase and buffer phase, respectively. The maximum absorption of the Soret band around 408.0 nm was used as the absorbance. The absorbance of proteins was determined with UV-vis spectrometer (UV-2550, Shimadzu Co., Kyoto, Japan).

The environment of heme vicinity of cyt.*c* in the IL phase was determined by Resonance Raman (RR) Spectroscopy. The Raman spectra were obtained on a JASCO NRS-1000 spectrometer with a Kaiser Optical holographic notch-plus filter and a liquid N_2_-cooled change-coupled device (CCD) detector. Data were accumulated for 150 s with spectral resolution of 4.0 cm^−1^. The excitation source was a Coherent Innova 90C Kr laser with a 20 mW beam at a 413.1 nm excitation wavelength.

The redox activity of cyt.*c* in the IL phase was determined by UV-vis spectrometer. An excess amount of sodium dithionite was added and stirred gently. The absorption of cyt.*c* in the IL phase before and after addition of the sodium dithionite was determined by UV-vis spectroscopy.

### 3.6. Extraction of cyt.*c* Dissolved in IL Phase to Aqueous Phase

[C_4_mim][Tf_2_N] and P_555_C4S were mixed as the mixing ratio of [C_4_mim][Tf_2_N]/P_555_C4S as 1:1. Cyt.*c* was dissolved into phosphate buffer (100 mM, pH 7.0). The aqueous solution of cyt.*c* (2 mg mL^−1^) was added to [C_4_mim][Tf_2_N]/P_555_C4S mixture as 1:10 by volume of IL:buffer solution. The resulting solutions were stirred vigorously. Then, the concentration of K_2_HPO_4_/KH_2_PO_4_ salts was set to 400 mM by adding high concentration buffer solution (1000 mM) to the mixture. The environment of heme vicinity and redox activity of cyt.*c* extracted from IL phase to buffer phase in the mixture was determined by RR spectroscopy and UV-vis spectroscopy, respectively.

## 4. Conclusions

In conclusion, we have used 1-butyl-3-methylimidazolium bis(trifluoromethanesulfonyl)imide as a hydrophobic IL to form phase separated state with aqueous buffer solution. The saturated water content of the hydrophobic IL phase was considerably improved from 0.4 wt% to 62.8 wt% by adding *N*,*N*,*N*-tripentyl-4-sulfonyl-1-butanephosphonium-type zwitterion. The biphasic system containing 62.8 wt% water in IL phase successfully dissolved *horse heart* cytochrome *c* (cyt.*c*) without significant change of the higher ordered structure. The saturated water content of IL phase was controlled by the concentration of inorganic salt of buffer solution. Cyt.*c* dissolved in IL phase was extracted again from IL phase to aqueous phase by controlling the concentration of inorganic salt in the mixture without significant change of the higher ordered structure.
